# Arthroscopic internal drainage and cystectomy of popliteal cyst in knee osteoarthritis

**DOI:** 10.1186/s13018-017-0670-4

**Published:** 2017-11-23

**Authors:** Jun Jiang, Lei Ni

**Affiliations:** Arthritis Clinic & Research Center, Peking University People Hospital, #11 Xizhimen South Avenue, Xicheng District, Beijing, 100044 China

**Keywords:** Popliteal cyst, Osteoarthritis, Knee, MRI, Arthroscopy

## Abstract

**Background:**

The purpose of this study was to evaluate the efficacy of arthroscopic knee cavity internal drainage and cyst cavity debridement operation of popliteal cyst in knee osteoarthritis patients.

**Methods:**

From August 2007 to March 2013, 58 knee osteoarthritis patients with popliteal cyst were treated with arthroscopic knee cavity internal drainage through posteromedial portal and popliteal cyst cavity debridement through superior posteromedial portal. In all patients, preoperative magnetic resonance imaging (MRI) was performed to detect combined intra-articular pathology and the communication between popliteal cyst and knee cavity. Clinical efficacy was evaluated through VAS score and Lysholm score.

**Results:**

All patients had neither recurrence of popliteal cyst nor complaints of pain, swelling, or functional impairment at average 24 months follow-up after surgery. Postoperatively, VAS score was decreased significantly and Lysholm score was raised significantly comparing preoperatively.

**Conclusion:**

Arthroscopic knee cavity internal drainage operation through posteromedial portal and popliteal cyst cavity debridement through superior posteromedial portal is an effective minimally invasive surgery method for the treatment of popliteal cyst without recurrence in knee osteoarthritis patients.

## Background

A popliteal cyst, originally called Baker’s cyst, is a synovial fluid-filled mass located in the popliteal fossa. The most common synovial popliteal cyst is considered to be a distension of the bursa located between the medial head of the gastrocnemius muscle and semimembranosus muscle. Usually, in an adult patient, popliteal cyst is complicated with knee osteoarthritis [1].

Some scholars thought that popliteal cyst in knee osteoarthritis patients is communicated with knee cavity and closely related to joint effusion [2]. When joint effusion is reduced, popliteal cyst will disappeared automatically and need not to be excised [3]. But our clinical experience shows that clinical result of conservative treatment for popliteal cyst in knee osteoarthritis patients is not good enough.

Usually, popliteal cyst in knee osteoarthritis patients will be excised through open surgery from posterior popliteal fossa by most orthopedic surgeon. Some scholars suggest that popliteal cyst can be arthroscopically excised directly from posterior popliteal fossa [4]. But both methods have high risk of injury to popliteal nerves and vessels and have high recurrence. Moreover, open surgery has cosmetic problems because of large incision scar [5].

Popliteal cysts have been shown to be complicated with knee osteoarthritis and have unidirectional synovial fluid valvular mechanism from knee cavity to popliteal cyst [6, 7]. Arthroscopic knee cavity internal drainage (posteromedial capsulotomy between the medial head of gastrocnemius and semimembranosus muscle through posteromedial portal) will cause bidirectional synovial fluid flow mechanism between knee cavity and popliteal cyst. The correction of the unidirectional valvular mechanism would prevent recurrence of popliteal cyst. When joint effusion in knee osteoarthritis is reduced, popliteal cyst will shrink, even disappear gradually. So there is no need to perform open surgery. To facilitate popliteal cyst shrinking, arthroscopic cystectomy was also performed through superior posteromedial portal. Simultaneously, intra-articular lesion of knee osteoarthritis should be addressed and popliteal cyst cavity debridement through superior posteromedial portal should be performed arthroscopically.

The purpose of this study was to evaluate clinical efficacy of arthroscopic knee cavity internal drainage through posteromedial portal and cystectomy through superior posteromedial portal of popliteal cyst in knee osteoarthritis patients.

## Methods

### Clinical data of patients

From August 2007 to March 2013, 58 knee osteoarthritis patients with popliteal cyst were treated with arthroscopic knee cavity internal drainage through posteromedial portal and cystectomy through superior posteromedial portal. These patients include 18 men and 40 women. The average age was 63.5 years old (range 48~79 years). These popliteal cysts were located in the left knee in 26 patients and in the right knee in 32 patients. The average course of disease was 24 months (range 18~60 months).

Inclusion criteria was primary osteoarthritis with popliteal cyst.

The exclusion criteria were as follows: posttraumatic knee osteoarthritis patients with popliteal cyst, knee rheumatic arthritis patients with popliteal cyst, inflammatory arthritis (gout, pseudogout, psoriasis) patients with popliteal cyst, and popliteal cyst patients after total knee arthroplasty.

**Fig. 1:**
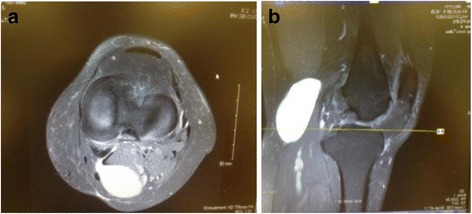
Preoperative MRI image of the left knee popliteal cyst. **a** Axial view shows that the popliteal cyst is located between the medial gastrocnemius and semimembranosus, and there is communication between popliteal cyst and knee cavity. **b** Sagittal view shows that the popliteal cyst is located posteriorly to the medial gastrocnemius

To evaluate the intra-articular lesions, preoperative magnetic resonance imaging (MRI) was performed for all patients. MRI showed that popliteal cyst was located between the medial head of gastrocnemius and semimembranosus muscle and there was communication between popliteal cyst and knee cavity (Fig. 1).

The indication for the arthroscopic operation included an MRI-detected popliteal cystic lesion, mass swelling in the popliteal fossa, pain and limitation of ROM (range of motion), and symptoms associated with an intra-articular lesion of knee osteoarthritis.

### Surgical procedure

**Fig. 2:**
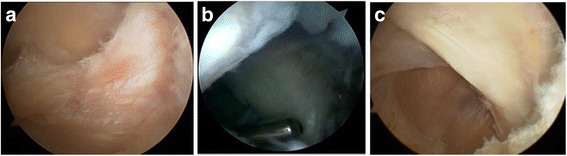
**a** An arthroscopic view through anteromedial portal shows the posteromedial capsular fold in front of popliteal cyst in the left knee osteoarthritis patient. **b** An arthroscopic view through anteromedial portal shows knee cavity internal drainage of popliteal cyst through resecting posteromedial capsule between the medial gastrocnemius tendon and semimembranosus tendon to produce bidirectional flow of popliteal cyst synovial fluid between knee cavity and popliteal cyst. The medial gastrocnemius tendon can be clearly observed. **c** An arthroscopic view through posteromedial portal shows popliteal cyst cavity (posteromedial to the medial gastrocnemius tendon)

**Fig. 3:**
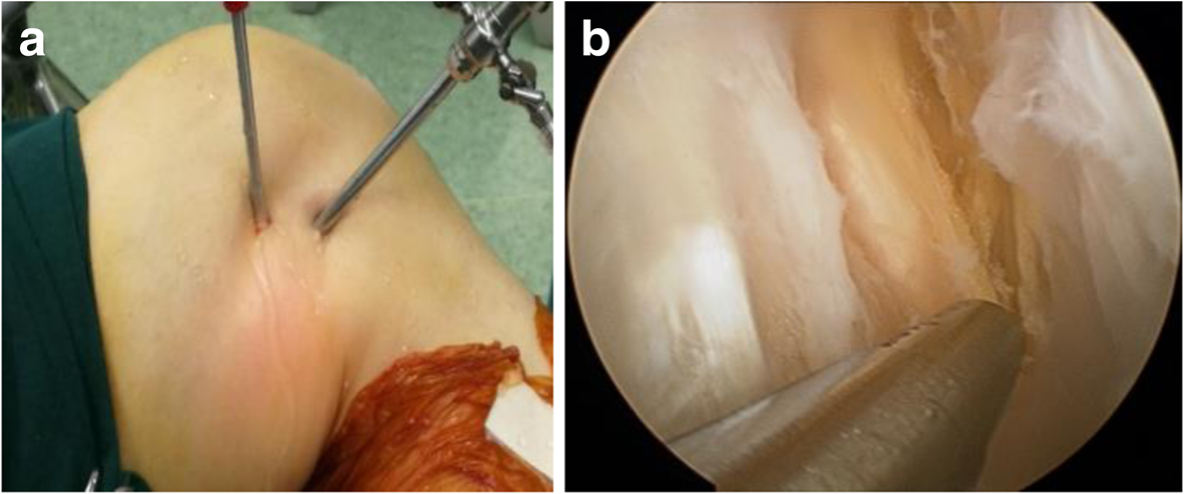
**a**, **b** An arthroscopic view through posteromedial portal shows debridement of synovial membrane (on the right side of the shaver) of popliteal cyst cavity working through superior posteromedial portal. The semimembrane tendon, gracilis tendon, and semitendinosus tendon aligned from anterior to posterior on the left side of shaver

These patients underwent arthroscopic surgery with spinal anesthesia in the supine position with tourniquet. Routine arthroscopic examination of the knee was performed through anterolateral (AL) portal. Arthroscopic debridement was performed to treat knee osteoarthritis including chondral lesion debridement, partial meniscectomy, and synovectomy through anteromedial (AM) portal. The arthroscope was redirected into posteromedial compartment from AL portal (or AM portal) through intercondylar notch between posterior cruciate ligament (PCL) and lateral wall of medial condyle with the knee at 60° flexion. We observed the posteromedial capsule and inserted spinal needle into posteromedial articular cavity within the boundary composed of medial collateral ligament (MCL), medial gastrocnemius, and semimembranosus with knee at 90° flexion, and then, we incised the skin and established a posteromedial (PM) portal. A probe was inserted to find the communication to the cyst. Usually, it is not easy to find the communication from articular cavity to the cyst. But there is always posteromedial capsular fold in front of popliteal cyst (Fig. 2a). The posteromedial capsular fold was resected with shaver through PM portal to thoroughly enlarge the communication between medial gastrocnemius tendon and semimembranosus tendon and to produce bidirectional flow of synovial fluid between knee cavity and popliteal cyst. The medial gastrocnemius tendon should be clearly revealed (Fig. 2b). Then, knee cavity internal drainage of popliteal cyst was accomplished arthroscopically. After this procedure, the arthroscope was transferred to PM portal to view popliteal cyst cavity (Fig. 2c). Under the view, with the knee bent to figure 4, a superior posteromedial (SPM) portal was established. Through SPM portal, synovial membrane of popliteal cyst cavity was debrided to reveal the semimembrane tendon, gracilis tendon, and semitendinosus tendon from anterior to posterior (Fig. 3). This procedure is useful for adhesion of medial gastrocnemius tendon and semimembrane tendon to close popliteal cyst cavity. Associated intra-articular lesions, such as meniscus tear, chondral lesions, and synovitis, were then treated with corresponding arthroscopic procedure, such as meniscectomy, chondral lesion debridement, and synovectomy. Once a drain was inserted into the knee cavity through PM portal, a compressive dressing should be applied in the popliteal fossa. Operation time was about 30 min to 1 h.

### Postoperative rehabilitation

Patients were encouraged to perform active knee flexion and isometric quadriceps muscle strength exercises on the second day after surgery which should be performed for about 3 months postoperatively. The drain was removed 2 days after surgery. Then, the patients were allowed to take partial weight bearing with crutch for about 6 weeks. And full weight bearing was permitted 6 weeks postoperatively.

### Clinical evaluation

In all patients, preoperative MRI was performed to detect intra-articular lesions and the communication between knee cavity and popliteal cyst. The patient was followed up postoperatively. We used VAS score and Lysholm score for clinical evaluation. The VAS score and Lysholm score were statistically compared between postoperatively and preoperatively with Student’s *T* test.

## Results

### Symptoms and signs

Preoperatively, popliteal cyst in knee osteoarthritis patients can cause discomfort and swelling symptoms. Some patients may feel weakness of the lower limb. When popliteal cyst was enlarged, it will influence range of motion. The knee joint could not be fully flexed or extended. The follow-up results postoperatively show that the aforementioned symptoms disappear.

Preoperatively, cystic mass, swelling, fluctuation, tension, and local tenderness in the popliteal fossa can be felt through physical examination. The follow-up results postoperatively show that the aforementioned signs disappear and no recurrence.

### Preoperative MRI examination

Medial meniscus lesions were mostly found in 55 patients (grade III mild lesion). Chondral lesion in medial compartment and patellofemoral joint were totally found in all patients because of knee osteoarthritis. Some patients also had synovitis and hydrarthrosis.

### VAS pain score and Lysholm knee function score

The follow-up period was average 24 months (18~60 months). Clinical efficacy of the surgical procedure was evaluated through VAS pain score and Lysholm knee function score. The VAS pain score was decreased from preoperative 8.8 (± 3.5) points to postoperative 3.1(± 1.4) points. The Lysholm knee function score was raised from preoperative 43.6 (± 5.3) points to 87.5 (± 3.7) points. Both have statistical significance through Student’s *T* test, shown in Table 1.

**Table 1:** VAS and Lysholm score $\left(n=58\kern1em \overline{X}\pm S\right)$

	VAS score	Lysholm score
Preoperative	8.8 ± 3.5	43.6 ± 5.3
Postoperative	3.1 ± 1.4	87.5 ± 3.7
*T* value	10.96	78.53
*P* value	0.0000	0.0000

## Discussion

Knee osteoarthritis is a chronic degenerative disease in adults with the main pathology of cartilage degeneration and osteophyte, accompanied with meniscus tear, synovitis, and loose body. In adults, popliteal cysts usually occur concomitantly with knee osteoarthritis, which results in persistent and excess production of synovial fluid [8, 9].Through the study of 426 knees, Labropoulos et al. [10] found that the incidence rate of popliteal cyst increased with the age, especially for the population older than 50 years. Marti-Bonmati et al. [11] studied the prevalence of popliteal cyst through MRI examination. They found 145 popliteal cysts in 382 knees (38%) and that there was extremely significant association between knee joint effusion and popliteal cyst (*P* = 0.002). There was a significant association between meniscal injury and popliteal cyst (*P* = 0.01).

Most scholars agree that popliteal cyst is a distention of the bursa located between the medial head of the gastrocnemius muscle and semimembranosus. The bursa space is communicated with knee cavity through one-way valvular mechanism covered by posteromedial capsular fold. Synovitis and chronical joint effusion in knee osteoarthritis will increase articular cavity pressure and can cause continuous unidirectional flow of synovial fluid from articular cavity to popliteal cyst because of low pressure in popliteal cyst. The synovial fluid in popliteal cyst could not return to knee articular cavity. So the popliteal cyst will be enlarged gradually.

Sansone and De Ponti [7] thought that medial meniscus lesion was the key of popliteal cyst formation. There was medial meniscus lesion in 84–90% of popliteal cyst patients. They thought that posterior horn tear of medial meniscus could act as valvular mechanism between knee articular cavity and popliteal cyst. But Takahashi and Nagano [12] had suspect on the theory. They put arthroscope into posteromedial compartment through posteromedial portal and found there was big distance between posterior horn of medial meniscus and communication of popliteal cyst with knee articular cavity. So it was impossible for posterior horn tear of the medial meniscus to act as valvular mechanism between knee articular cavity and popliteal cyst. Takahashi found “cranny structure” of posteromedial capsule observed in arthroscopic surgery can act as valvular mechanism.

According to our clinical data, there was no obvious “cranny structure” in posteromedial capsule. Maybe the “cranny structure” in posteromedial capsule is very tiny to observe arthroscopically. But posteromedial capsular fold can always be observed arthroscopically, which may be self-closure of communication between popliteal cyst and knee cavity to prevent its continuous enlargement.

There are two arthroscopic surgery methods to disrupt unidirectional flow of popliteal cyst in knee osteoarthritis patients. Communication enlargement is posteromedial capsule fold resection to produce bidirectional flow of synovial fluid between popliteal cyst and knee cavity. Gradually, popliteal cyst will disappear because of the elimination of synovial fluid in popliteal cyst. Communication closure is arthroscopic suture of posteromedial capsular fold suture to close unidirectional flow of synovial fluid [13]. The success rates were 96.7 and 84.6% in the communication enlargement group [7, 14–18]and communication closure group [12, 19, 20], respectively. Communication enlargement was subgrouped into cystectomy wall resection group [14, 15, 17, 18] and the non-cystectomy wall resection group [7, 16], for which the success rates were 98.2 and 94.7%, respectively.

We performed partial posteromedial capsulotomy (communication enlargement between articular cavity and popliteal cyst) to produce enough bidirectional flow of synovial fluid (arthroscopic articular cavity internal drainage of popliteal cyst). We also performed cystectomy wall debridement through superior posteromedial portal, which will be useful for popliteal cyst cavity closure through adhesion of medial gastrocnemius tendon and semimembranosus tendon. The treatment effect of technically easily performed communication enlargement is definitive than that of communication closure, because communication closure is not definitive to close the communication and more technically difficult to perform.

It is very necessary to perform MRI examinationpreoperatively. MRI is helpful to find not only the communication between knee articular cavity and popliteal cyst but also the underlying intra-articular lesions, such as meniscus lesion, synovitis, and chondral lesion. The popliteal cyst is almost never an isolated pathology in adult knee.

Since a significant association of Baker’s cysts with knee joint disorders has been reported, treatment should primarily address articular lesions causing recurrent effusions. Arthroscopic surgery provides an effective treatment in that both the cyst and associated joint disorders can be optimally visualized and accordingly simultaneously treated [13].

An open surgical excision from posterior popliteal fossa cannot be considered as a definitive solution in most popliteal cyst patients. The frequency of recurrence, large popliteal skin scars, and limitation of knee motion after simple open surgical excision from posterior popliteal fossa lead to arthroscopic excision internal drainage of popliteal cyst through posteromedial portal.

Arthroscopic surgery has several benefits: relatively simple, minimally invasive, and early rehabilitation. And most importantly, it is just to excise the posteromedial capsular fold which will enlarge valvular communication to lead to bidirectional flow of synovial fluid, and cystectomy wall can also be arthroscopically resected and performed through superior posteromedial portal.

We did not perform MRI examination after arthroscopic knee articular cavity internal drainage of popliteal cyst and cystectomy cyst wall debridement. The main reason is that the arthroscopic knee cavity internal drainage of popliteal cyst is just to enlarge the communication between knee cavity and popliteal cyst. There is still some remnant fluid in the popliteal cyst. But the pressure inside the popliteal cyst will decrease and the symptoms will disappear. Through cystectomy cyst wall debridement, popliteal cyst cavity will disappear gradually through adhesion of medial gastrocnemius tendon and semimembranosus tendon. So there is no need to perform MRI examination postoperatively.

Our study has proved that arthroscopic internal drainage and cystectomy of popliteal cyst in knee osteoarthritis is effective minimally invasive surgical procedure without recurrence, which was shown by decreased VAS pain score and increased Lysholm knee function score and no recurrence during follow-up period.

## Conclusion

Arthroscopic articular cavity internal drainage and popliteal cystectomy cavity debridement of popliteal cyst and arthroscopic debridement of knee osteoarthritis may eliminate popliteal cyst and improve knee function and is an effective minimally invasive surgery method for the treatment of popliteal cyst without recurrence in knee osteoarthritis patients.

## Abbreviations

ACRC: Arthritis Clinic & Research Center
AL: Anterolateral
AM: Anteromedial
JJ: Jun Jiang
LN: Lei Ni
MCL: Medial collateral ligament
PCL: Posterior cruciate ligament
PM: Posteromedial
SPM: Superior posteromedial
